# Understanding the scale and nature of avoidable healthcare-associated harm for prisoners in England: protocol for a retrospective cross-sectional study

**DOI:** 10.1136/bmjopen-2024-085607

**Published:** 2024-12-20

**Authors:** Andrew Carson-Stevens, Isobel Joy McFadzean, Thomas Purchase, Sioned Gwyn, Stuart Hellard, Kate Davies, Darren M. Ashcroft, Anthony Avery, Stephen Campbell, Adrian Edwards, Sandra Flynn, Thomas Hewson, Saied Ibrahim, Melanie Jordan, Richard N. Keers, Tim Millar, Maria Panagioti, Caroline Sanders, Jane Senior, Caroline Stevenson, Ellie Thompson, Florian Walter, Carl de Wet, Verity Wainwright, Jenny Shaw

**Affiliations:** 1 Division of Population Medicine, Cardiff University, Cardiff, Cardiff, UK; 2 Wales Centre for Primary and Emergency Care Research (PRIME Centre), School of Medicine, Cardiff University, Cardiff, Cardiff, UK; 3 The University of Manchester, Manchester, UK; 4 Patient Safety Research Collaboration (PSRC), National Institute for Health and Care Research, Manchester, UK; 5 NIHR Greater Manchester Patient Safety Research Collaboration (PSTRC), University of Manchester, Manchester, UK; 6 Centre for Academic Primary Care, School of Medicine, University of Nottingham, Nottingham, UK; 7 Centre for Pharmacoepidemiology and Drug Safety, Division of Pharmacy and Optometry, The University of Manchester, Manchester, UK; 8 Division of Population Health, Health Services Research & Primary Care, The University of Manchester, Manchester, UK; 9 Department of Public Health Pharmacy and Management, School of Pharmacy, Sefako Makgatho Health Sciences University, Pretoria, South Africa; 10 Centre for Mental Health and Safety, Division of Psychology and Mental Health, School of Health Sciences, Faculty of Biology, Medicine and Health, The University of Manchester, Manchester, UK; 11 Health Education England North West School of Psychiatry, Liverpool, Merseyside, UK; 12 School of Sociology and Social Policy, University of Nottingham, Nottingham, Nottinghamshire, UK; 13 Centre for Pharmacoepidemiology and Drug Safety Research, The University of Manchester, Manchester, UK; 14 Optimizing Outcomes with Medicines Research Unit, Pennine Acute Hospitals NHS Trust, Ashton-under-Lyne, UK; 15 Division of Nursing, Midwifery and Social Work, School of Health Sciences, The University of Manchester, Manchester, UK; 16 South West Hospital and Health Service, Gold Coast, Queensland, South West Hospital and Health Service, Roma, Queensland, Australia; 17 Greater Manchester Mental Health NHS Foundation Trust, Manchester, UK

**Keywords:** Patients, Prisons, Cross-Sectional Studies, Safety

## Abstract

**Abstract:**

**Introduction:**

Around 1 in 20 patients experience avoidable healthcare-associated harm worldwide. Despite longstanding concerns, there is insufficient information available about the safety of healthcare for prisoners. To address this, this study will investigate the scale and nature of avoidable healthcare-associated harm for prisoners in England.

**Methods:**

We will undertake a large retrospective cross-sectional study involving a case note review of patient healthcare records in 18 prisons in England. Prisons will be purposively sampled for maximum variation of characteristics based on prison category (open, local, training, high security, female), type (publicly and privately run) and prison population size, to sample approximately 15 000 patient records. We will focus on two samples: an enhanced risk sample of prisoners, considered to be at the most risk of healthcare-associated harm, and a random sample of prisoners excluded from the enhanced risk sample, to estimate the incidence of avoidable harm, and express this as ‘per 100 000 patients per year’. Avoidable harms will be characterised by type of incident(s), contributory incident(s), contributory factor(s), outcome(s) and severity of harm, prior to a thematic analysis of the relationships between those variables. Univariable and multivariable analyses will be conducted to identify factors associated with avoidable harm.

**Ethics and dissemination:**

The decision regarding participation by prisons within the study will be voluntary, and their consent to participate may be withdrawn at any time. We will not seek individual patient consent for the retrospective case note review of their records, but if patients respond to publicity about the project and inform us that they do not wish their records to be included, we will adhere to their wishes. We will produce a report for the Department of Health’s Policy Research Programme and several peer-reviewed publications. The study has been granted a favourable opinion by Wales Research Ethics Committee 3 (reference 19/WA/0291), Her Majesty’s Prison and Probation Service (reference 2019–332) and the Confidentiality Advisory Group (CAG) to access the medical records without individual consent under Section 251 of the National Health Service Act 2006 (reference 19/CAG/0214).

**STRENGTHS AND LIMITATIONS OF THIS STUDY**

- The study will estimate the scale and nature of avoidable healthcare-associated harm for prisoners.
- A purposive sample of 18 prisons stratified by type, population size and geography will produce a diverse sample of adult prisons in England to generate transferable findings.
- Case note review relies on the ability of a clinical reviewer to extract evidence based on what is, and is not, stated in the medical records, drawing from their clinical knowledge and awareness of guidelines. We will conduct sensitivity analyses to estimate whether any avoidable harm experienced by patients is missed.
- The rate of harm ‘per 100 000 patients per year’ will represent harm or potential harm that occurs when people receive care during their time residing in prison, and therefore some harm may not be detected due to the nuance of secure envionments, or will only become apparent after they leave prison.

## Introduction

###  Background/rationale

There is long-standing concern regarding healthcare provision for people who reside in prisons, herein named prisoners, globally.^1^ A review of health outcomes in secure and detained settings highlighted the need to improve service responsiveness at the interface between prison regime and healthcare.^2 3^ Healthcare outside of prisons is fraught with challenges to deliver safe care, and a meta-analysis of 147 international studies found that 5% of patients experience avoidable harm, and 14% of these harms cause permanent disability or death.^4^ None of the included studies were from the prison context. While some prison-based studies have focused on issues like self-harm,^5^,^7^ wider evidence on the scale, nature and severity of avoidable harm does not yet exist for prison healthcare.^4^

Prison is a challenging environment to provide health services due to the complex needs of the population.^8^ The number of people in prison in England and Wales currently stands at approximately 80 000, with over 60% of prisons experiencing overcrowding.^9^ Women make up just 4% of the prison population across 13 prison establishments^9^ but have a higher prevalence of poor physical and mental health in comparison to the general population, often preceded by childhood trauma, and their healthcare needs are not being met in prison.^10^ Prisoners can be a socioeconomically disadvantaged group, with multiple health morbidities including substance use, and chronic cardiopulmonary conditions manifesting at a younger age than the general population.^2^ Over 90% of prisoners experience at least one mental health condition, including common mental health problems, severe and enduring mental illness, personality disorders, and/or substance use.^2 5 11^ Older adults are now the fastest growing group in prisons in England and Wales and have high demands for health and social care services.^10 12^ Prisoners commonly have chaotic and unsettled lives, with sporadic engagement with routine healthcare in the community, and an increased use of expensive non-routine services, such as emergency departments.^13^

The National Health Service (NHS) Health and Justice commissioning teams aim to adhere to the principle of ‘equivalence’ whereby prisoners should receive the same services and standard of care as the rest of the population to achieve equitable health outcomes.^8^ However, the demand for services, and lack of adequate resources, raises concerns that prisons may only be able to focus healthcare provision to patients with the greatest need.^14^ A retrospective multimethod analysis of prison patient safety incident reports highlights problems with accessing healthcare professionals, medication and poor staffing levels,^15^ and the Nuffield Trust has identified issues with both healthcare access and safety.^16 17^

There are several challenges which should be considered when planning a study to investigate avoidable healthcare-associated harm for prisoners. Healthcare is provided predominately by primary care teams, but many patients receive additional services, including secondary care, psychiatry, substance use treatment services, dentistry and obstetric/maternity care.^3 8 18^ Attending hospital or outpatient departments is reliant on the availability of prison personnel to accompany them as escorts. Continuity across health and justice providers is variable, and transferring medical records and data between healthcare settings is challenging, with healthcare teams finding it difficult to share information and deliver personalised care for prisoners.^16^ While some improvements have been made in recent years, there are still concerns that prisons are unable to meet the standards of care expected within the community, and reports have shown poor communication between healthcare professionals working in prisons and prison officers.^18^ There are also apprehensions that commissioning practices in prisons are ‘siloed’, with organisations working in isolation, and care being delivered by different statutory agencies and/or funded by different governmental and healthcare departments.^19^ This has led to confusion within referral pathways regarding what funding streams are available, and this is exacerbated by moving and transferring prisoners between the jurisdictions of Boards/Trusts as they are moved between prisons.

Using healthcare records to inform assessment of care quality and safety, known as case note review, can inform epidemiological estimates of healthcare-associated harm, which has been conducted in multiple settings, including primary care.^20^ Multiple sources of data exist within electronic medical records, for example, from the notes kept by the primary care team (and additional service providers), to information about investigations ordered, actions taken by the reviewing professionals and correspondance to and from secondary care. In combination, they will provide the opportunity to assess the safety of care provided for prisoners, and not just from the prison healthcare team alone.

### Aims

We will explore the scale and nature of avoidable patient harm for prisoners receiving healthcare using case note review.

### Objectives

- Estimate the incidence of avoidable healthcare-associated harm for prisoners.
- Quantify, describe and classify the nature and severity of avoidable patient harm.
- Identify contributing factors that, if addressed, could reduce the incidence of avoidable harm in prisons.

## Methods

### Study design

The study began in June 2019 and will be completed in November 2024. In keeping with previous studies, to inform estimates, we will undertake a retrospective cross-sectional study involving case note review of the healthcare received by prisoners within a 12-month census period.^20^ We will identify those at highest risk of healthcare-associated harm (our ‘enhanced sample’), using two independent healthcare professionals (doctors/nurses) to screen medical records, and we will assess the inter-rater reliability of their judgements.^21^ Records of those included in the enhanced sample, as well as a sensitivity analysis of those not included, will be reviewed by general practitioners (GPs).^3 22^ We will also use an established scale to assess the avoidability of harm in the primary care context,^21 23^ that is, harm averted by prevention, or is amenable, for example, by treatment.^24^

Our definitions of harm and avoidability^25^ are shown in box 1. Further, our reviewers will be trained to consider acts of omission as well as commission, if they believe that it has contributed to the outcome of healthcare-associated harm. For example, they should report events in which healthcare teams did not prescribe, dispense or administer medication, as well as when the incorrect medication has been prescribed, dispensed or administered.

Box 1 **Definition of avoidable harm^24^**

A patient safety incident could have been probably, or totally, avoided by the timely intervention of a healthcare professional (e.g., investigations, treatment, safety netting) and/or an administrative process (e.g., referrals, alerts in electronic health records, procedures for following up results) in accordance with accepted standards of evidence-based practice and/or clinical governance and/or the Bolam test*.

*The Bolam test refers to whether a healthcare professional can show that they acted in a reasonable and defendable way that a responsible body of healthcare professionals in the same field would regard as acceptable, taking into account evolving standards of care.^23^

Harm can be either physical or psychological and reviewers will judge potential cases of avoidable harm against this definition, and consider whether they are satisfied to state that ‘the staff/prison could have done no more’.

The judgement about avoidability will be made on a two-tier basis:

- *Tier 1:* appraisal of whether more could have been done as per care delivery within the community (ie, embracing the concept of equivalence of care to achieve equitable health outcomes). A judgement will be made about avoidability without consideration of any caveats introduced by the prison regime, system and environment.
- *Tier 2:* with prison-experienced healthcare professional input, a further appraisal of whether or not the prison could have done more, considering any restrictions imposed by the regime and environment (eg, resources, service availability and a lack of prisoner autonomy to coordinate appointments).

The GP reviewers will be asked to use this concept of care equivalence and will be prompted to indicate their initial impressions of avoidability, relating to primary care, secondary care and the prison regime. To ensure consistency, final avoidability judgements will be made by the study team, which includes prison-experienced healthcare professionals who will support Tier 2 judgements.

### Setting

18 prisons in England.

### Eligibility of prisons for entering the study

#### Inclusion criteria

Prisons in England will be eligible to participate if:

- They provide Governor approval.
- They deliver onsite healthcare services to prisoners.
- They have electronic health records, and use the electronic healthcare system SystmOne,^26^ which >90% of healthcare facilities^27^ in the prison estate uses.

### Recruitment of prisons and data collectors

We will review all prisoner medical records/case notes (estimated approximately 15000) in the participating prisons. A purposive sample of 18 prisons is required and will exhibit maximum variation across important characteristics including prison category (open, local, training, high security, female), type (publicly and privately run), population size and geography, that will provide transferable findings about the scale, nature and avoidability of harm in prison healthcare. Prison healthcare provider will also be reviewed (ie, whether NHS or privately commissioned), but as providers are predominately private, we will not sample on this basis alone. The complexities of commissioning, subcontracting healthcare, and specific approvals for research within the NHS will inform our purposive and pragmatic sampling decisions.

Independent nurses and doctors will be recruited and trained to undertake data screening, and GPs will be recruited and trained to undertake data collection from prison electronic medical records. Doctor and GP reviewers will each have greater than 5 years of experience and will not have been excluded from the General Medical Council register. The nurse reviewers will be registered general nurses or registered mental health nurses, and they must not have been excluded by the Nursing and Midwifery Council. We will actively seek sufficient numbers of nurses, doctors and GPs to meet data collection requirements, and while prison experience will be encouraged, it will not be made mandatory.

We will encourage retention of the prisons in the study by ensuring that the data collection procedures are not disruptive to the workings of the healthcare or prison teams. Nurse and doctor screeners, and GP reviewers will have remote access to the medical records and will receive payment for their work on the project.

### Data sources

We will access SystmOne electronic clinical record systems, which contain detailed demographic and health information on all prisoners.

### Sampling approach

**Figure 1:**
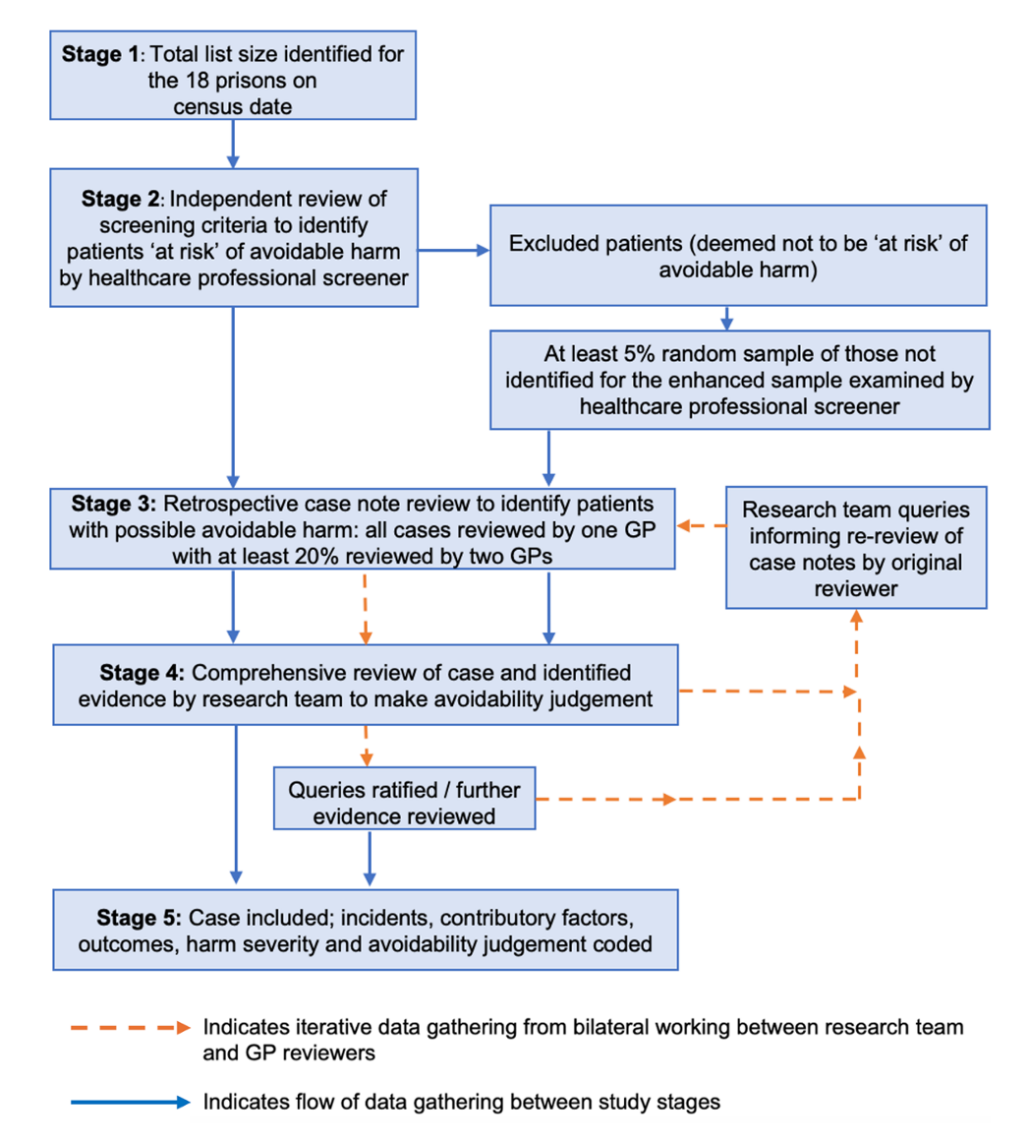
Stages of the study and flow of data gathering throughout the study.

We will employ the following comprehensive five-stage approach to screening and reviewing the medical records of cases eligible for inclusion in our analysis (see figure 1).

For each prison, we will include records for all people in prison present on a specific census day (stage 1). The census period for data collection will vary by prisons owing to differing timelines for establishing contact and getting prisons onboard for the study, and are between 26 December 2020 and 12 July 2021, and we will consider this variation in our analyses.

To capture anyone who would still have been in the prison had they not died, we will also ask HM Prison and Probation Service to notify us of any deaths that occurred in the year prior to the census date, and decide whether records would be included or not (see online supplemental file 1 appendix 1). We have estimated that after accounting for turnover, there will be approximately 15000 patient records across the 18 participating prisons (comprising of approximately 12% of the prison population in England overall).

#### Developing enhanced sampling criterion to detect those at higher risk of harm

In our study of significant avoidable harm in primary care, the incidence of harm for patients without ‘enhanced sample’ risk factors was low.^20^ To identify prisoners at increased risk of avoidable harm (‘enhanced sample’) (Stage 2), nurses or doctors will screen all medical records in the participating prisons (approximately 15 000). Screening will include reviewing records for up to 12 months prior to the census day (fewer if the prisoner was only in the prison for a shorter duration), and the number of months of data available for each prisoner will be recorded to calculate ‘patient years’ as the denominator for our main outcome measure.

We expect that the enhanced sample criteria used in our primary care study^20^ will result in a relatively small number of cases of avoidable significant harm, due to differences between prisons and the wider general population (eg, age, prevalence of multi-morbidity, i.e. the co-existence of two or more healthcare conditions for the same individual^28^ and frailty). In addition, we seek to identify evidence of all severities of harm, whereas the primary care study focused on significant harms only. For this reason, we will have a lower threshold in terms of the detection of prisoners in the enhanced risk sample than in the primary care study.

Drawing on the expertise accrued from leading the similar study in primary care in England,^20^ prior working in prison contexts,^15 25^ our knowledge from similar studies to identify high-risk patients from primary care populations,^29^ and taking into account specific circumstances or triggers in which episodes of avoidable harm may be noted, we have developed a list of enhanced sample criteria for the prison population, including:

- A new significant health problem as defined in our primary care study^20^ .
- Multimorbidity.
- Allergies or adverse drug reactions.
- Polypharmacy (>10 regular medications).
- Admission to an Emergency Department or an unscheduled admission to hospital/a mental health facility.
- Acute health problem following substance use.
- Died during the study period.
- Did not attend scheduled hospital appointments.
- Required attention from an out of hours healthcare professional or paramedic.
- Abnormalities in the following blood results:
  - Urea and electrolytes: eGFR change of ≥5 in patients with an eGFR <45 (ie, chronic kidney disease stage 3b).
  - Aspartate aminotransferase or alanine aminotransferase >150.
  - Haemoglobin <100 g/L.

We anticipate that this ‘low threshold’ approach wil provide more cases for in-depth analysis.

#### Pilot screening using enhanced sample criteria

In our primary care study, ~15% of patients were categorised into the enhanced risk sample.^20^ In the prison population, we expect that the prevalence of certain clinical presentations will be higher (eg, acute health problems following substance use), although the population is younger. We initially estimated that this will result in up to 2250 prisoners in the ‘enhanced risk sample’. Given the absence of prior studies from the prison context to inform our estimates, in the early stages of the study in April 2022, we carried out a pilot screening of 794 cases in a single prison to review the frequency of each criterion, seeking to observe any clustering of eligibility criteria, and critically judge cases to inform our enhanced sample selection.

Based on our eligibility criteria to screen for those ‘at risk’ of avoidable harm, we found 375 (47%) to be part of the enhanced sample. The proportion of patients screened who met the eligibility criteria ranged from between 1% and 20%. The most common eligibility criteria met were not attending scheduled hospital appointments, followed by multimorbidity and significant health problems (table 1). Overall, 174 (46%) of the 375 cases in the enhanced sample met two or more criteria. As the prevalence in the prison was much higher than in the primary care study, we estimate that up to 7000 of 15 000 records will be screened into the enhanced sample for full case record review by a GP.

**Table 1:** Frequency and proportions of patients meeting each eligibility criterion

Eligibility criterion for the enhanced sample	N	%
Did not attend scheduled hospital appointments	157	20%
Multimorbidity	119	14%
A new significant health problem	114	14%
Admission to an Emergency Department or an unscheduled admission to hospital or a mental health facility	107	13%
Required attention from an out of hours medic or paramedic	100	13%
Polypharmacy (>10 regular medications)	44	6%
Allergies or adverse drug reactions	29	8%
Abnormalities in blood results	14	2%
Acute health problem following substance use	13	2%
Died during the study period	7	1%
**Totalenhanced sample (any of the above criteria)**	**375**	**47%**

### Inter-rater reliability for stages 2 and 3

To assess inter-rater reliability of the screening by research nurses or doctors, a random sample of at least 5% will be screened by a second screener for every prison (stage 2). Overall agreement and Cohen’s Kappa statistic (with 95% CI) will be estimated for each eligibility criterion.

All records within the enhanced sample will have an independent review by one GP, and a random sample of up to 20% of records will have a re-review by a second GP to identify possible avoidable harm (stage 3). Like stage 2, we will assess inter-rater reliability using a Cohen’s Kappa statistic (with 95% CIs). In addition, a random sample of at least 750 records not selected for the ‘enhanced sample’ will have a review by two GPs, to identify possible avoidable patient harm.

This comprehensive review process will enable us to draw valid judgements on the likelihood that avoidable healthcare-associated harm has occurred.

#### Sample size

We aim to review all case notes in the participating prisons (estimated approximately 15 000). As this is the first study of its type in prison, a range of potential incidences and precision of avoidable harm with sufficient power (>80%) was based on a sample of 15 000 prisoners (patients), as shown in table 2. These estimates are informed from a study in English primary care, and allow for higher than expected rates of certain avoidable harms in prisons (eg, self-harm).^21^

**Table 2:** Sample size calculations for incidence of avoidable harm

Estimated incidence (per 100 000 patient years)	Precision based on an adjusted sample size of ~15 000 (95% CI)
100	4.6 (5.4 to 14.6)
150	5.6 (9.4 to 20.6)
200	6.5 (13.5 to 26.5)
500	10 (40 to 60)
1000	15 (85 to 115)
2000	20 (180 to 220)
5000	31 (469 to 535)

#### Data collection

For any cases of harm the GPs consider to be avoidable, pseudo-anonymised data will be entered onto a bespoke Case Report Form (stage 4, summarised in box 2). This will be completed electronically via a secure, encrypted link to the study database (see online supplemental file 1 appendix 2).

Box 2 **Data collection**

The general practitioners reviewing the healthcare records will detail the following:

- A free-text narrative recording any new healthcare problems within the census period, any evidence of self-harm and any experience of significant health problems within the prison.
- A free-text narrative describing the way that the health problem could have potentially been prevented by healthcare (primary care, secondary care or prison specific).
- Up to five medications specifically involved in the incident, if applicable.
- The severity of the harm.
- The avoidability of the harm and justification for their chosen avoidability rating.

The study team will then review the submitted cases and record:

- The ‘primary incident’ defined as the patient safety incident that occurs proximally to the patient experiencing a harm-related outcome.
- Up to three ‘contributory incidents’ defined as incidents that contribute to the occurrence of another incident (including the primary incident).
- Up to four ‘contributory factors’ defined as the circumstances, actions or influences which play a part in how an incident originates / develops, or increases the risk of a patient safety incident.
- Up to four harm-associated outcomes defined as ‘the impact on a patient which is wholly or partially attributable to an incident’.
- The severity and avoidability of the harm

Our approach is designed to generate informative descriptive summaries and understand the relationships between important concepts like incident type (ie, what happened) and contributory factors (ie, the tasks and the processes commonly underpinning the issues raised). These relationships highlight important opportunities to improve patient safety.^30^

Reviewers will judge the ‘avoidability’ of harm on a 6-point scale, ranging from totally unavoidable to totally avoidable,^21^ considering the influence of primary care, secondary care and the prison system. The data collection form will encourage reviewers to explain events in their own words, informed by evidence they will identify, and why they believe that patient harm has occurred.

#### Primary and secondary outcomes

The primary outcome will be the rate of patient harm judged at least probably avoidable. The secondary outcome will be the rate of patient harm judged at least possibly avoidable.

#### Classification of avoidable harm cases

We will use the comprehensive PatIent SAfety (PISA) classification system to deconstruct the case narratives written by the GP reviewer(s) to record the type of patient safety incident, contributory incidents, contributory factors and severity of harm.^30^ The research team will support GP reviewers to use a recursive incident analysis approach^31^ to describe the events leading up to the patient safety incident which resulted in harm.^30^ Fortnightly, quality assurance teleconference meetings will be held with the GP reviewers to achieve ongoing clarity about the identified evidence.^32^ Where necessary, reviewers will be required to re-review notes to identify further evidence, or confirm the absence of information that may be relevant to the case.

### Consistency of judgements regarding avoidable harm

Each report will be reviewed in conjunction with relevant research evidence (eg, National Institute for Health and Care Excellence and Scottish Intercollegiate Guidelines Network guidelines) to support decision-making about the ‘avoidability’ of the incident(s) that led to healthcare-associated harm.^13 21^ If evidence is not available, we will apply the Bolam test; that is, to apply the standards, we believe practitioners would be held to by a responsible body of medical opinion. The research team will discuss disagreements, and if there is any uncertainty, the case will be referred to the chief investigator (CI) for a final decision.

**Figure 2:**
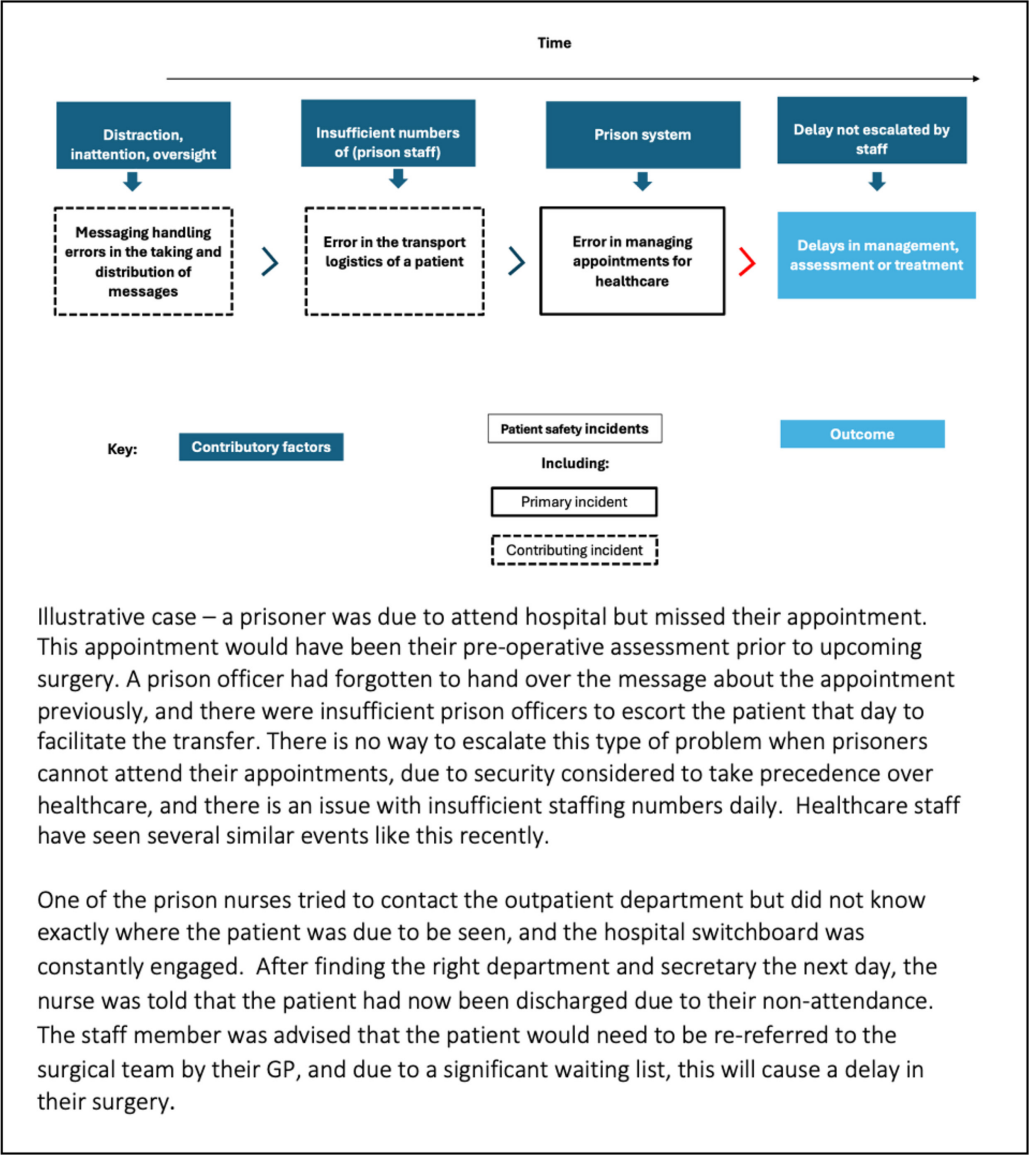
Example of an illustrative case coded using the PISA classification system, adapted from McFadzean *et al*.^15^

We will also identify the chronological sequence of events leading up to an incident by drawing on the recursive model for incident analysis, to allow us to build a structured and coded sequence, using codes from several classification frameworks (eg, see figure 2).

By reviewing the case in figure 2, it is possible to understand the apparent incidents that led to harm and the contributory factors/incidents. Using the coded sequences for further analysis, higher-level patterns in the data across cases will be explored by examining the frequencies of the relationships between, and combinations of, incidents and contributory factors. These analyses will enable us to identify potential priority issues to mitigate risk/enhance patient safety.

### Bias

#### Sampling of practices and patients

To enhance transferability of findings, we will recruit a purposive sample of 18 prisons. All prisoners in those establishments will be eligible for inclusion, although bias may be introduced if prisoners with more serious conditions object to a review of their records. As noted above, to address the possibility that patients with avoidable harm are not included in the enhanced risk sample, a random sample of up to 5% of ‘stage 1’ patients that are not screened into the enhanced sample (‘stage 2’) will be re-screened. To address the possibility that GP reviewers ‘miss’ prisoners with avoidable harm when reviewing the stage 3 sample, a random sample of up to 20% of these records will be re-reviewed by a second GP reviewer.

#### Training of and support for reviewers

GP reviewers involved in the retrospective case note review will need to adhere to consistent methods for identifying patients with avoidable harm. To reduce reviewer bias, a blended learning programme has been developed for professionals participating in stages 2 and 3, including three asynchronous pre-recorded lectures and an online panel discussion with prison-based healthcare professionals. The three lectures cover (i) the role and duties of reviewers during the study, explanation of Human Factors in healthcare and principles of patient safety incident analysis; (ii) a description of the state of prison healthcare in the UK, a comparison between prison-based healthcare services and community-based primary care, and an orientation to key documentation used in prisons; and (iii) example scenarios (based on real cases) of patient safety incidents in prisons.

All identified cases of avoidable harm will be reviewed independently by at least two members of the study team (academic GPs: IJM TP SG and ACS). The findings of each independent review will be compared to determine the extent to which the reviewers (study team members and GP reviewers) agree on the identification and classification of avoidable harm. Disagreements will be monitored in real time and discussed in regular teleconferences, to ensure appropriate detail is gathered, whilst adhering to our definitions of harm. These calls will also allow the GP reviewers to bring difficult cases for peer discussion.

## Analyses

### Descriptive analysis

We will summarise the demographics of the prisoners (eg, age, gender) and prisons (type and size). We will make comparisons across the prison estate in England to establish transferability (size, type and geography) and estimate the incidence of avoidable harm ‘per 100 000 patient years’ accompanied with 95% confidence intervals. To account for the high turnover, we will use the number of case notes available over a 12-month period, multiplied by the length of stay in the specific prison, as a denominator.

### Statistical analysis

Univariable regression analyses will be conducted to identify factors (relating to prisoners and prisons) associated with avoidable healthcare-associated harm. Statistically significant risk factors (at the 10% significance level to avoid missing important risk factors) will be assessed by multivariable analysis. We will also assess interrater reliability of judgements made by paired reviewers using the Kappa statistic (with 95% CI) for stages 2 (nurse and doctor screening), and 3 (GP review) judgements. We will undertake risk prediction using regression models to identify the most important eligibility criteria that are considered risk factors that contribute to cases of avoidable harm. A sensitivity analysis of the incidence rate of avoidable harm identified, and the risk factors associated with them, in the sample not included in the ‘enhanced sample’ will also be examined.

### Qualitative analysis

To ensure sufficient detail within the reporting to support the feasibility of qualitative analysis, we will quality assure the narratives as they are submitted by reviewers, prompting them to provide clarifying information and encouraging additional information as needed. We will meet with the GP reviewers two times per month to promote detailed reporting. The quality assurance process is guided by the WHO’s Conceptual Framework for the International Classification of Patient Safety.^33^

From the descriptive analysis, we will identify patterns of avoidable patient harm by the relationship(s) between types of patient safety incident, contributory incident(s), contributory factor(s) and outcome(s) (including severity of harm). These patterns will form the basis of our purposive sample of reports with similar characteristics, for example, the most frequently observed relationships, the most severe outcomes experienced by patients and the most and least avoidable incidents.^30 34^

As per previous studies, we anticipate that a thematic analysis will permit a more nuanced and in-depth appreciation of contextual insights not captured by our initial coding process.^15 20^ Emergent themes will be inductively developed to capture the cross-cutting nature of systemic failures resulting in common avoidable harm outcomes, and in turn will support our recommendations for safety improvement. The study team will review and refine the codes and themes to iterate a narrative that explains our findings. We will use our knowledge of community-based care and the concept of care equivalence to deepen our understanding of clinical situations within prisons and aim to further contextualise, understand and interpret what has been reported, with our stakeholders.

## Ethics

The NHS Research Ethics Committee approval, Confidentiality Advisory Group support and Her Majesty’s Prison and Probation Service (HMPPS) approval have been obtained.

Governor approval will be sought from each establishment via a letter of introduction. Enclosed within this correspondance will be a briefing document with further details on what the study involves, as well as a copy of the HMPPS approval. Prisons will be asked to confirm their participation in the study in writing and to provide a specific point of contact (SPOC) for the study. If a response is not received, the research team will follow-up with two reminders, via email and telephone. Prisons will be substituted with a suitable alternative where necessary.

The research team will discuss the set-up of the study with each SPOC, and provide the prison with posters (including opt-out information), study promotion information (eg, a summary of the study) to ensure prisoners are aware of the study, and that they can opt-out if they do not wish for their medical records to be reviewed. Study promotion will continue for at least six weeks before data collection commences (as per our primary care study).^21^ If, during this time or subsequently, any prisoner decides to opt-out of the study, the prison healthcare team will add a code to their medical records confirming that they do not wish their medical records to be used. Those prisoners will not be included on case lists for reviewers. In addition, where nurse, doctor and GP data collectors see this code on the medical records, they will know not to review their data as part of this study.

Should any GP reviewer identify any ongoing concerns for prisoners during the case note review, the study team will notify the healthcare provider of this so that they can act as needed. For example, in some circumstances, the healthcare team may decide to take further action to protect prisoners, or to inform prisoners about problems that have occurred with their care. In extreme circumstances, if the reviewers, or the Chief Investigator (CI) of the study, have ongoing concerns that the healthcare provider are not taking appropriate action, they may relay their concerns to the relevant Responsible Officer for each prison. Only cases where GP reviewers have concerns about ongoing care will be flagged to healthcare providers, as opposed to examples of substandard care or evidence of avoidable harm that have already occurred.

### Patient and public involvement (PPI) representatives

The PPI representatives gave input to the Confidentiality Advisory Group and Ethics application; notably, our decisions about consent-seeking processes. They are also members of the project management group, will convene at least monthly, and will be encouraged to actively contribute to discussions about the conduct of the study. They will also contribute to a minimum of one of the early meetings with GPs who will be involved in data collection, so that they can provide their views on the operationalisation of definitions of avoidable harm.

## Dissemination

We will disseminate the research findings using a wide range of approaches, including a plain English summary, conference presentations, publications (in a range of formats) and stakeholder events.

For policymakers, we will meet with relevant colleagues within the Department of Health and Social Care (DHSC), NHS England, Ministry of Justice (including Safer Custody) and relevant Arm’s-Length Bodies at key points during the project. We will discuss the conduct of future assessments of the scale and nature of avoidable harm in prison healthcare, and will advise on interventions for reducing the incidence of these harms. We will share emergent findings during the project. Together with a Service User Group and a Stakeholder Advisory Group, we will explore methods of further dissemination, including holding a stakeholder event and running webinars.

For the public, we will suggest to the DHSC to approach the Science Media Centre (www.sciencemediacentre.org/) to launch the findings of the study.

For the prisons and the healthcare providers involved in the project, we will provide feedback on the findings of the study, particularly focusing on practical suggestions for improving patient safety and reducing the incidence of avoidable harm in prison healthcare.

For clinicians, academics and policymakers, we will present the findings of our research at conferences and will publish in high-quality, peer-reviewed journals. In keeping with the approach from our ‘avoidable harm in primary care’ study,^21^ we will work closely with relevant government and professional bodies (eg, Ministry of Justice and relevant Arm’s-Length Bodies, Royal College of General Practitioners) to disseminate strategies for reducing the incidence of avoidable harm.

## supplementary material

10.1136/bmjopen-2024-085607 online supplemental file 1

10.1136/bmjopen-2024-085607 online supplemental file 2
