# Supplementary material for: Understanding the scale and nature of avoidable healthcare-associated harm for prisoners in England: protocol for a retrospective cross-sectional study
Source: BMJ Open. 2024 Dec 20;14(12):e085607. doi: 10.1136/bmjopen-2024-085607 (PMC11667317; doi:10.1136/bmjopen-2024-085607)
Supplement: online supplemental file 1 [file bmjopen-14-12-s001.pdf]

APPENDIX 1

Notification of deaths within the census period by His Majesty's Prison and Probation Service (HMPPS)

To reliably include a death in our screening list, we need to ascertain whether the deceased would have been in one of in the included prisons on a given census date, had they not died.

Information requested from HMPPS includes, by prison:

- Name
- Date Of Death
- Prison number
- Index offence
- Status (e.g., sentenced or remand)
- Sentence date (if applicable)
- Sentence length (if applicable)

Sentenced prisoners

Inclusion criteria:

- Imprisonment for public protection (IPP) sentences, Lifer status, on a recall or sentence lengths of 4 or more years (and therefore subject to parole).
- For sentence lengths of less than 4 years, calculate half of sentence length to ascertain whether they would have been in prison on the census date and include/exclude on this basis.
- A limitation to acknowledge is that we will have to assume the prisoner would not have transferred to another prison before our census date, but this will likely be very small numbers.

Remand prisoners

Include all.

Although we have no way to calculate possible release date / outcome of hearings, the remand prisoner group is under-represented in prison research and experience exclusion in varying forms more generally. By including all and being able to filter these cases when it comes to analysis, we will be able to capture information about this population whilst acknowledging they may not have been in our sample. By also capturing the number of remand deaths we are notified of, we will also be able to make a reasoned statement regarding how much of a limitation this is within the data (i.e., acknowledging number across how many of our prisons given that some of our selection will not house remand prisoners at all).

APPENDIX 2

DATA COLLECTION FORM

Question	Data option	Answer
Date of case note review:	Date selection	
Patient ID:	Already in place	
Prison ID:	Already in place	
Patient age at start of census period:	Input numeric	
Gender of patient:	Select from dropdown	Male, Female, Other, Non-Binary, Not stated
Has there been a new health problem during the 12-month review period?	Select from dropdown	Yes, No
Input a read code to describe the nature of the health problem	Free text	
Is there any evidence of self-harm?	Select from dropdown	Yes, No
Is there evidence that the patient has an active ACCT document?	Select from dropdown	Yes, No
Did the patient receive an adequate standard of care for this health problem based on your review of the case notes? If YES STOP CODING:	Select from dropdown	Yes, No
Where an adequate standard of care is not apparent for the health problem, has the patient experienced healthcare associated harm?	Select from dropdown	Yes, No
Did the problem(s) in care occur exclusively in the prison?	Free text	
Did it start in another prison?	Select from dropdown	Yes, No
Did it start in the community?	Select from dropdown	Yes, No
How many transfers have they had in the census year?	Select from dropdown	0 through to 10

How many prison transfers?	Select from dropdown	0 through to 10
How many community to prison transfers?	Select from dropdown	0 through to 10
Was the outcome potentially avoidable?	Select from dropdown	Yes, No
What was the actual harmful outcome that the patient experienced?	Free text	
What was the severity of the harmful Outcome 1?	Select from dropdown	Caused death, or brought death forward in the short term, on the balance of probabilities.<br><br>Caused a major long-term or permanent impact on physical, mental, or social functioning. Required prolonged hospitalisation or admission to HDU/ITU. Required major medical or surgical intervention (most often delivered in a hospital setting).<br><br>Caused severe or long-term psychological/emotional distress to the patient. Required hospitalisation (not prolonged) or treatment in A&E, not requiring major intervention.<br><br>Caused a medium-term (>1 month) impact on physical, mental, or social functioning.<br><br>Caused a mild and short- term impact, on physical, mental, or social functioning, that was expected to resolve in a few hours.<br><br>Was harmed but required no or minimal intervention/treatment, e.g., anti-emetic, oral antibiotic or repeat of a minor procedure such as vaccination or insertion of contraceptive implant.<br><br>Experienced transient emotional distress but no long-term consequences and medical records report contains words, e.g., angry, anxious, confused, distressed, frightened, frustrated, humiliated, or upset, that might describe a feeling that occurs at the time of the incident but soon passes.<br><br>No Harm (EXCLUDE AS NOT A CASE OF AVOIDABLE HARM)
What was the severity of the harmful Outcome 2?	Select from dropdown	See options for Outcome 1 above.

What was the severity of the harmful Outcome 3?	Select from dropdown	See options for outcome 1 above
On a scale of 1-6 where 1 = totally unavoidable and 6 is totally avoidable how avoidable was the outcome from a primary care perspective?	Select from dropdown	1 (Definitely not preventable by Primary Care)<br>2 (Slight evidence for preventability by Primary Care)<br>3 (Possibly preventable by primary care, but less than 50-50)<br>4 (Probably preventable by Primary Care, more than 50-50 but close call)<br>5 (Strong evidence for preventability by Primary Care)<br>6 (Definitely preventable by Primary Care) Exclude
From a prison perspective was the outcome potentially avoidable?	Select from dropdown	Yes, No
On a scale of 1-6 where 1 = totally unavoidable and 6 is totally avoidable how avoidable was the outcome?	Select from dropdown	See options for primary care perspective above
From a secondary care perspective was the outcome potentially avoidable?	Select from dropdown	Yes, No
On a scale of 1-6 where 1 = totally unavoidable and 6 is totally avoidable how avoidable was the outcome?	Select from dropdown	See options for primary care perspective above
Describe the patient safety incident(s) that led to the harmful outcome and provide details of any events leading up to the incident(s). Be explicit about what evidence from the records is available. Provide details about what went wrong chronologically (include dates / timelines to document the time between the incident and the outcomes). Where there has been more than one case of avoidable harm, please number these in your narrative and we will create multiple forms for this patient since avoidability assessments will be needed for each case of avoidable harm:	Free text	
Please describe how the health problem and associated avoidable harm might have been prevented or made less serious by others (including primary care professionals, prison staff, secondary care, healthcare professionals or the patient). Where possible, differentiate events / factors concerning care by each professional group:	Free text	

For health problem 1 and related harm, how many transfers occurred whilst they experienced the issue?	Header	
HP1 how many prison to prison transfers?	Select from dropdown	0 through to 20
HP1 how many community to prison transfers?	Select from dropdown	0 through to 20
For health problem 2 and related harm describe how many transfers occurred whilst they experienced the issue?	Header	
HP2 how many prison to prison transfers?	Select from dropdown	0 through to 20
HP2 how many community to prison transfers?	Select from dropdown	0 through to 20
For health problem 3 and related harm describe how many transfers occurred whilst they experienced the issue?	Header	
HP3 how many prison to prison transfers?	Select from dropdown	0 through to 20
HP3 how many community to prison transfers?	Select from dropdown	0 through to 20
How might care have differed in the community setting?	Free text	
Is this an interesting case?	Select from dropdown	Yes No
Any issues with the completeness of the medical record?	Select from dropdown	Yes No
What data is missing from the medical record that would allow you to judge avoidability with more accuracy?	Free text	
Could you benefit from discussion with key personnel at the prison re: their processes / protocols / procedures?	Select from dropdown	Yes No
Reviewer's additional notes:	Free text	
Reviewer allocation:	Already in place	
Observations by clinical team:	Header	
Queries raised from clinical discussions:	Free text	

Review complete for coding:	Select from dropdown	Yes No
Areas for clarification from GP reviewer	Header	
GP rereview comments:	Free text	
Have you read the Cardiff reviewer's feedback:	Select from dropdown	Yes No
Record updated please select a date:	Enter date	
