# Supplementary material for: Understanding the scale and nature of avoidable healthcare-associated harm for prisoners in England: protocol for a retrospective cross-sectional study
Source: BMJ Open. 2024 Dec 20;14(12):e085607. doi: 10.1136/bmjopen-2024-085607 (PMC11667317; doi:10.1136/bmjopen-2024-085607)
Supplement: Supplementary file 3 [file bmjopen-2024-085607.reviewer_comments.pdf]

PEER REVIEW HISTORY

BMJ Open publishes all reviews undertaken for accepted manuscripts. Reviewers are asked to complete a checklist review form and are provided with free text boxes to elaborate on their assessment. These free text comments are reproduced below.

ARTICLE DETAILS

Title (Provisional)

Understanding the scale and nature of avoidable healthcare-associated harm for prisoners in England: protocol for a retrospective cross-sectional study

Authors

Carson-Stevens, A; McFadzean, Isobel Joy; Purchase, Thomas; Gwyn, Sioned; Hellard, Stuart; Davies, Kate; Ashcroft, Darren; Avery, Tony; Campbell, Stephen M; Edwards, Adrian; Flynn, Sandra; Hewson, Tom; Ibrahim, Saied; Jordan, Melanie; Keers, Richard Neil; Millar, Tim; Panagioti, Maria; Sanders, Caroline; Senior, Jane; Stevenson, Caroline; Thompson, Ellie; Walter, Florian; de Wet, Carl; Wainwright, Verity; Shaw, Jennifer

---

VERSION 1 - REVIEW

**Reviewer**	**1**
**Name**	**Bellass, Sue**
**Affiliation**	**Manchester Metropolitan University**
**Date**	**19-Mar-2024**
**COI**	**None.**

Thank you for the opportunity to review this excellent study protocol, which details plans for an ambitious study that will make an important contribution to the knowledge base on prison healthcare safety.

To further strengthen the paper I would like to suggest a few areas that would benefit from minor revisions.

1. Please could you include information on your study timelines and some indication of the anticipated number of reviewers.

2. Page 8, line 58 - Page 9, line 4. It would be helpful to readers unfamiliar with the structure of prison healthcare provision to expand on the issues you allude to concerning care continuity and the impact of siloed commissioning.

3. Related to the point above, I wondered whether prison healthcare provider would be a factor in your purposive sampling of the 18 prisons.

4. Page 10, box 1. The text relating to Tier 2 is not visible.

5. Page 13, line 56. Definitions of multimorbidity vary. It would be helpful if you could give more details on what you would classify as multimorbidity apart from the stated number of up to 6 conditions.

6. I note the case note reviewers are independent GPs or nurses, but it's not clear to me whether they have experience of prison healthcare. I assume you are not viewing prison healthcare experience as essential given you are providing some training on the prison context, but given they will be expected to make a judgement on whether the harm was potentially avoidable in the prison setting (data collection form, page 37) could you offer a brief consideration of how the reviewers' backgrounds may influence the rating scale data?

7. Please could you provide more information on how you plan to conduct the qualitative analysis (page 22) in a manageable yet rigorous way, given the scale of the task? The amount of free text in the data collection tool that may be subject to qualitative analysis will be significant, especially when considering the likely number of case notes that will be reviewed.

Thank you again for the opportunity to review the paper and I wish the team the best of luck with the study.

**Reviewer**	**2**
**Name**	**Fajreldines, A**
**Affiliation**	**Hospital Alemán, Calidad y seguridad del paciente**
**Date**	**20-Mar-2024**
**COI**	**None**

This study is a outstanding contribution. It also fully describes the topic under discussion. I can contribute with three comments, which I hope will clarify further the matter. 1-Training details and skills acquired by data collectors should be fully specified, so it may become clear how to replicate this process in every location. 2-When there are events originated from omissions in medical care, would those events be considered or not? . 3-when it comes to preventability of events due to drug mechanisms, preventability assessment tools and/or the experts who participate in such assessments should have a different and specific tool or matrix to evaluate events. This tool or matrix must be different from other health adverse events assessment tools.

VERSION 1 - AUTHOR RESPONSE

Comments

Reviewer: 1: Dr. Sue Bellass, Manchester Metropolitan University
Thank you for the opportunity to review this excellent study protocol, which details plans for an ambitious study that will make an important contribution to the knowledge base on prison healthcare safety.
To further strengthen the paper I would like to suggest a few areas that would benefit from minor revisions.
Thank you again for the opportunity to review the paper and I wish the team the best of luck with the study.

Reviewer: 2: Dr. A Fajreldines, Hospital Aleman
This study is a outstanding contribution. It also fully describes the topic under discussion. I can contribute with three comments, which I hope will clarify further the matter.

Reviewer	Comment	Response
Reviewer 1	1. Please could you include information on your study timelines and some indication of the anticipated number of reviewers.	Thank you. The study began in June 2019 and will be completed by November 2024; the Census periods for data collection varied dependent on prison recruitment and are between 26/12/2020 and 12/07/2021.<br><br>We are now close to completing data collection and do not envisage recruiting any more staff; to-date, this includes two nurse reviewers and 15 GP reviewers.<br><br>The study timeline and the following text has been added to the methods section:<br><br>"GPs reviewers will each have greater than five years of practice experience and will not have been excluded from the General Medical Council register. The nurse reviewers will be registered general nurses (RGNs), or registered mental health nurses (RMNs) and they must not have been excluded by the Nursing and Midwifery Council. We will actively seek sufficient numbers of nurses and GPs to meet data collection requirements, and whilst prison experience will be encouraged, it will not be made mandatory."
	2. Page 8, line 58 - Page 9, line 4. It would be helpful to readers unfamiliar with the structure of prison healthcare	Thank you for your suggestion. We have amended the text to read:<br><br>Continuity across health and justice providers is variable, and transferring medical records and data between healthcare settings is challenging, with healthcare teams finding it difficult to share

	provision to expand on the issues you allude to concerning care continuity and the impact of siloed commissioning.	information and deliver personalised care for prisoners[16]. Whilst advances and improvements have been made in recent years, there are still concerns that prisons are unable to meet the equivalence of care expected within the community, and reports have shown poor communication between healthcare professionals working in prisons and prison officers[18]. There are also apprehensions that commissioning practices in prisons are "siloed", in which organisations work in isolation, care is delivered by different statutory agencies and commissioned by different governmental and healthcare departments[19]. This has led to confusion with referral pathways and related funding available from commissioners, exacerbated by the movement and transfer of prisoners between jurisdictions of boards / Trusts as they are moved between prisons.<br><br>References<br>16. Nuffield Trust. *How prison health care in England works* 2021; Available from: https://www.nuffieldtrust.org.uk/resource/prison-health-care-in-england.Accessed Dec 2023. -<br>18. Hayton, P. and J. Boyington, *Prisons and health reforms in England and Wales*. American Journal of Public Health, 2006. 96(10): p. 1730-1733.<br>19. Justice Committee., *Prison Population 2022: Planning for the future.* London: House of Commons, 2019.
	3. Related to the point above, I wondered whether prison healthcare provider would be a factor in your purposive sampling of the 18 prisons.	Thank you. We have amended the text within the section 'Recruitment of prisons and data collectors':<br><br>"Prison healthcare provider will also be reviewed, (i.e. whether NHS or privately commissioned) but as providers are predominately private, we will not sample on this basis alone. The complexities of commissioning, subcontracting of healthcare, and specific approvals for research within the NHS, will inform our purposive and pragmatic sampling decisions."
	4. Page 10, box 1. The text relating to	We apologise if you could not review all the text within the document we uploaded. It read:

	Tier 2 is not visible.	The judgement will be made on a two-tier basis:<br><br>▪ **Tier 1:** assessment of whether more could have been done, with equivalence to how community cases would be assessed. Therefore, a judgement is made regarding avoidability, without consideration of the caveats introduced by the prison regime, system, and environment.<br>▪ **Tier 2:** With prison-experienced General Practitioner (GP) input, a further assessment should be made about whether or not the prison could have done more, considering the restrictions imposed by the regime and environment (i.e., to aid an understanding of what aspects of the prison environment may have contributed to harm cases [e.g., resource and service availability, lack of prisoner autonomy to coordinate appointments etc.]).<br><br>Harm will not be classified as unavoidable due to the prison setting alone and will be judged based on the Tier 1 context. The additional information gathered as part of Tier 2 will help understand prison specific constraints/explanations of the harm that may help inform policy."<br><br>We have also added the following sentences for clarity:<br><br>"The GP reviewers will be asked to use the concept of equivalence of care and will be prompted to indicate their initial impressions of avoidability from the perspective of primary care, secondary care and the prison regime. To ensure consistency, final avoidability judgements will be made by the study team, including prison-experienced GPs and other healthcare professionals, who will support Tier 2 judgements. Any rating disagreements between the reviewers and the study team will be

		discussed by the research team, and if disagreement remains, the case will be referred to the Chief Investigator (CI) for a final decision.”
	5. Page 13, line 56. Definitions of multimorbidity vary. It would be helpful if you could give more details on what you would classify as multimorbidity apart from the stated number of up to 6 conditions.	We have used the World Health Organization’s definition [28], as the co-existence of two or more health conditions for the same individual and have added this to the manuscript.<br><br>Reference<br><br>28. Mercer, S., Furler, J., Moffat, K., Fischbacher-Smith, D. and Sanci, L., 2016. *Multimorbidity: technical series on safer primary care*. World Health Organization.
	6. I note the case note reviewers are independent GPs or nurses, but it's not clear to me whether they have experience of prison healthcare. I assume you are not viewing prison healthcare experience as essential given you are providing some training on the prison context, but given they will be expected to make a judgement on whether the harm was potentially avoidable in the prison setting (data collection form, page 37) could you offer a brief consideration of how the	Thank you, whilst some of the reviewers will have prison experience, this will not be considered mandatory during recruitment. We want the reviewers to concentrate on equivalence of care and not be influenced by the prison context and will ask them to indicate initial impressions of availability by thinking about primary care, secondary care, prison regime.<br><br>We have added the following in the text:<br><br><u>Recruitment and data collectors</u><br><br>“GPs reviewers will each have greater than five years of practice experience and will not have been excluded from the General Medical Council register. The nurse reviewers will be registered general nurses (RGNs), or registered mental health nurses (RMNs) and they must not have been excluded by the Nursing and Midwifery Council. We will actively seek sufficient numbers of nurses and GPs to meet data collection requirements, and whilst prison experience will be encouraged, it will not be made mandatory.”<br><br><u>Box 1:</u><br><br>“The GP reviewers will be asked to use the concept of equivalence of care and will be prompted to indicate their initial impressions of

	reviewers' backgrounds may influence the rating scale data?	avoidability from the perspective of primary care, secondary care and the prison regime. To ensure consistency, final avoidability judgements will be made by the study team, including prison-experienced GPs and other healthcare professionals, who will support Tier 2 judgements. Any rating disagreements between the reviewers and the study team will be discussed by the research team, and if disagreement remains, the case will be referred to the Chief Investigator (CI) for a final decision.”
	7. Please could you provide more information on how you plan to conduct the qualitative analysis (page 22) in a manageable yet rigorous way, given the scale of the task? The amount of free text in the data collection tool that may be subject to qualitative analysis will be significant, especially when considering the likely number of case notes that will be reviewed.	Thank you, we have edited the text as follows*:*<br><br>“Qualitative analysis<br>To ensure sufficient detail within the reporting to support the feasibility of qualitative analysis, we will quality assure narratives as they are submitted by reviewers, prompting them to provide clarifying information and encouraging additional information as needed. We will meet with the GP reviewers twice monthly to promote detailed reporting. The quality assurance process is guided by the World Health Organization’s Conceptual Framework for the International Classification of Patient Safety [33].<br><br>From the descriptive analysis, patterns of avoidable patient harm will be identified by the relationship(s) between type of patient safety incident, contributory incidents, contributory factors, and outcomes (including severity of harm). These patterns will form the basis of our purposive samples of reports with similar characteristics, for example, in terms of the most frequently observed relationships, the most severe outcomes experienced by patients, and the most and least avoidable incidents [34, 35].<br><br>As per previous studies, we anticipate a thematic analysis will permit a more nuanced and in-depth appreciation of contextual insights not captured by our initial coding process [15, 20]. Emergent themes will be inductively developed to capture the cross-cutting nature of systemic failures resulting in common avoidable harm outcomes, and in turn will be the basis of our recommendations for safety improvement. The

		study team will review and refine the codes and themes, to iterate a narrative that explains our findings. We will use our knowledge of community-based care, and the concept of care equivalence to deepen our understanding of clinical situations within prisons and aim to further contextualise, understand and interpret what has been reported with stakeholders. "<br><br>References<br>15. McFadzean, I.J., et al., *Patient safety in prisons: a multi-method analysis of reported incidents in England*. Journal of the Royal Society of Medicine, 2023: p. 01410768231166138.<br>20. Avery, A.J., et al., Incidence, nature and causes of avoidable significant harm in primary care in England: retrospective case note review. BMJ Quality & Safety, 2021. 30(12): p. 961-976.<br>33. Runciman WB, Baker GR, Michel P*, et al* Tracing the foundations of a conceptual framework for a patient safety ontology *Quality and Safety in Health Care* 2010;**19:**e56.<br>34. Urquhart, A., et al., Learning from patient safety incidents involving acutely sick adults in hospital assessment units in England and Wales: a mixed methods analysis for quality improvement. Journal of the Royal Society of Medicine, 2021. **114**(12): p. 563-574.<br>35. Andrew, C.-S., et al., A cross-sectional mixed methods study protocol to generate learning from patient safety incidents reported from general practice. BMJ Open, 2015. **5**(12): p. e009079.
Reviewer 2	1-Training details and skills acquired by data collectors should be fully specified, so it may become clear how to replicate this process in every location.	Thank you, we have added to the text:<br><br>"GPs reviewers will each have greater than five years of practice experience and will not have been excluded from the General Medical Council register. The nurse reviewers will be registered general nurses (RGNs), or registered mental health nurses (RMNs) and they must not have been excluded by the Nursing and Midwifery Council. We will actively seek sufficient numbers of nurses and GPs to meet data collection requirements,

		and whilst prison experience will be encouraged, it will not be made mandatory.”
	2-When there are events originated from omissions in medical care, would those events be considered or not?	Thank you for your question, and we apologise if this was not clear, we have edited the text within the study design to read:<br><br>“Our reviewers will be trained to considers acts of omission as well as commission, if they believe that this has contributed to the outcome of healthcare associated harm. For example, they should report events in which healthcare teams did not prescribe, dispense or administer medication, as well as when the incorrect medications have been prescribed, dispensed or administered.”
	3-when it comes to preventability of events due to drug mechanisms, preventability assessment tools and/or the experts who participate in such assessments should have a different and specific tool or matrix to evaluate events. This tool or matrix must be different from other health adverse events assessment tools.	Thank you for your suggestion. For standardisation and comparison between healthcare related harm reports, we have designed the data collection form to include the same information for each healthcare related harm and consideration of avoidability has been the focus of both the Department of Health and Social Care and the World Health Organization.<br><br>Addition to the text<br><br>“The GP reviewers will be asked to use the concept of equivalence of care and will be prompted to indicate their initial impressions of avoidability from the perspective of primary care, secondary care and the prison regime. To ensure consistency, final avoidability judgements will be made by the study team, including prison-experienced GPs and other healthcare professionals, who will support Tier 2 judgements. Any rating disagreements between the reviewers and the study team will be discussed by the research team, and if disagreement remains, the case will be referred to the Chief Investigator (CI) for a final decision.”
